# Short‐term rapamycin treatment increases ovarian lifespan in young and middle‐aged female mice

**DOI:** 10.1111/acel.12617

**Published:** 2017-05-22

**Authors:** Xiaowei Dou, Yan Sun, Jiazhao Li, Jing Zhang, Dandan Hao, Wenwen Liu, Rui Wu, Feifei Kong, Xiaoxu Peng, Jing Li

**Affiliations:** ^1^ State Key Laboratory of Reproductive Medicine Nanjing Medical University Nanjing Jiangsu 211166 China; ^2^ College of Animal Science and Veterinary Medicine Heilongjiang Bayi Agricultural University Daqing Heilongjiang 163319 China

**Keywords:** aging, mTOR, ovary, rapamycin

## Abstract

Although age‐related ovarian failure in female mammals cannot be reversed, recent strategies have focused on improving reproductive capacity with age, and rapamycin is one such intervention that has shown a potential for preserving the ovarian follicle pool and preventing premature ovarian failure. However, the application is limited because of its detrimental effects on follicular development and ovulation during long‐term treatment. Herein, we shortened the rapamycin administration to 2 weeks and applied the protocol to both young (8 weeks) and middle‐aged (8 months) mouse models. Results showed disturbances in ovarian function during and shortly after treatment; however, all the treated animals returned to normal fertility 2 months later. Following natural mating, we observed prolongation of ovarian lifespan in both mouse models, with the most prominent effect occurring in mice older than 12 months. The effects of transient rapamycin treatment on ovarian lifespan were reflected in the preservation of primordial follicles, increases in oocyte quality, and improvement in the ovarian microenvironment. These data indicate that short‐term rapamycin treatment exhibits persistent effects on prolonging ovarian lifespan no matter the age at initiation of treatment. In order not to disturb fertility in young adults, investigators should in the future consider applying the protocol later in life so as to delay menopause in women, and at the same time increase ovarian lifespan.

## Introduction

Global life expectancy in 2015 has increased to 71.4 years, and most notably, women live longer than men in every country of the world, with a life expectancy of 73.8 years (WHO, 2016). However, compared with the other organs in the body, the female gonad—the ovary—ages exceptionally early and rapidly (Li *et al*., 2012). Menopause, at approximately 50 years of age, is considered to constitute the end of the ovarian lifespan (Broekmans *et al*., 2009). Then, accompanied by the loss of ovarian gametogenic function, the significant decrease in ovarian gonad steroids, especially estrogen, will result in hypoestrogenism in later life (Buckler, 2005). This means that a woman will spend her postmenopausal life with increasing risk of cardiovascular disease, vasomotor changes, osteoporosis, and cognitive dysfunction which, in many cases, significantly affect the quality of her later years (Buckler, 2005).

The decline in ovarian function with aging is characterized by gradual depletion of ovarian follicles and a reduced ability to produce oocytes competent for fertilization and further development (Tatone *et al*., 2008). For women, the size of the oocyte pool is set during intrauterine life and termed ovarian reserve, the evaluation of which could predict a woman's total fertility potential (Tremellen & Savulescu, 2014). Along with the depletion of oocyte numbers, oocyte quality also diminishes with increasing maternal age. It is believed that in aged females, the decrease in oocyte quality is due to the increase in meiotic nondisjunction, resulting in an increased rate of aneuploidy in the early embryo (Broekmans *et al*., 2009). Given the inexorable decline in female fertility with age, many strategies have been mentioned to delay ovarian aging by preserving follicle reserve and improving oocyte quality. It has been shown that ovarian function can be improved in aged females by chronic administration of pharmacologic doses of antioxidants such as resveratrol (Liu *et al*., 2013), and vitamins C and E (Tarin *et al*., 2002a) during the juvenile period and throughout adult reproductive life. However, these antioxidants exert significant long‐term negative effects on ovarian and uterine functions, leading to disruptions of estrous cycles, ovarian hypertrophy, alterations in sociosexual behavior, higher fetal death, and decreased litter size (Tarin *et al*., 2002b). Recent studies demonstrated that caloric restriction (CR) without malnutrition could prolong female fertility by preventing maternal age‐associated oocyte aneuploidy and meiotic spindle defects (Selesniemi *et al*., 2008, 2011). However, mice and rats maintained on CR showed reduced fertility or were completely infertile due to the inhibition of ovarian follicular development and irregular estrous cycles. In other words, the extension of reproductive lifespan occurred only when animals returned to normal diets (Tilly & Sinclair, 2013). Thus, we ask the question, is future clinical application at all practicable with such a sacrifice of reproductive capacity during a woman's childbearing years?

The mechanistic target of rapamycin (mTOR) is a serine/threonine protein kinase that functions as a master regulator of cellular growth and metabolism in response to nutrient and hormonal cues. mTOR functions in two distinct complexes, mTORC1 and mTORC2, depending upon each molecule's sensitivity to rapamycin. Rapamycin (Rapa), the inhibitor of mTORC1, has been shown to extend lifespan in yeast (Powers *et al*., 2006; Medvedik *et al*., 2007), nematodes (Robida‐Stubbs *et al*., 2012), fruit flies (Bjedov *et al*., 2010), and mice (Harrison *et al*., 2009), and to improve health span and metabolic functions in nonhuman primates (Ross *et al*., 2015). Studies have also provided a possible use of Rapa for the preservation of the primordial follicle pool and the prevention of premature ovarian failure (POF) (Adhikari *et al*., 2013). A recent study encompassing 10 weeks of Rapa treatment in rats revealed the possibility of delaying ovarian aging by suppressing primordial follicle activation and follicular development by modulating sirtuin signaling (Zhang *et al*., 2013). However, whether the treatment can prolong ovarian lifespan was not followed, and the treated mice experienced irregular estrous cycles and cessation of active fertility. The results suggest that similar detrimental side effects exist with regard to the reproductive capacity of treated animals. Therefore, if Rapa is to be used to delay ovarian aging, an appropriate protocol must be developed to overcome its side effects: on the one hand, we can choose to treat animals with a shorter duration in order to decrease as much as possible the damage to follicular development; conversely, we can choose to use Rapa later in life when fertility is not necessary for women. We hypothesize that Rapa prolongs ovarian lifespan, but it is still necessary to develop a practical protocol that has no serious side effects on fertility.

One recent study that provided new evidence of applications for Rapa suggested that a single 3‐month, but not lifelong, treatment increases life expectancy by up to 60%, and improved health span in middle‐aged mice (Bitto *et al*., 2016). Therefore, in the present study, we shortened the Rapa treatment to 2 weeks and applied it to adult female mice at two different ages (8 weeks and 8 months). The two ages represent humans at ages 20–30 and 38–47, respectively (The Jackson Laboratory). Our new protocol showed a transient disturbance in ovarian function during and shortly after Rapa treatment. However, follicular development and ovulation were restored to normal 2 months later, and ovarian lifespan was prolonged in both models. Our results provide data to support using Rapa in a transient manner that extends reproductive capacity, and the treatment can be started later in life.

## Results

### Part I. Assessment of 2‐week rapamycin treatment on ovarian lifespan in an 8‐week‐old (8 weeks) mouse model

#### Disturbances in ovarian function during a 2‐week treatment of rapamycin in a young, adult mouse model

**Figure 1:**
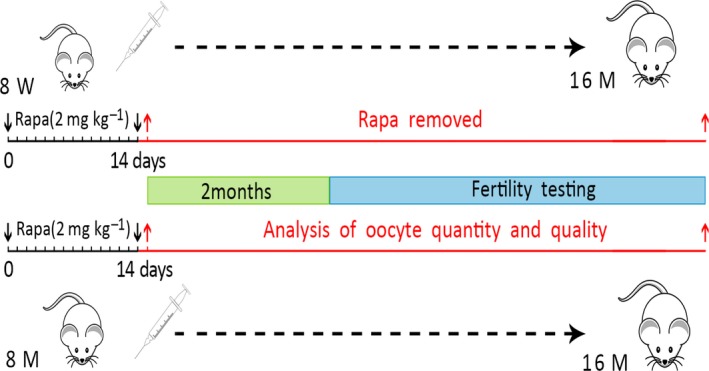
Two different models were established in the experiment: female mice at 8 weeks or 8 months of age were treated with Rapa (rapamycin, 2 mg kg^−1^) daily for 2 weeks. The drug was then removed and ovarian function was analyzed at indicated time points (red arrows), with endpoints being oocyte quantity and quality. Fertility testing was performed 2 months later and up to 16 months of age.

**Figure 2:**
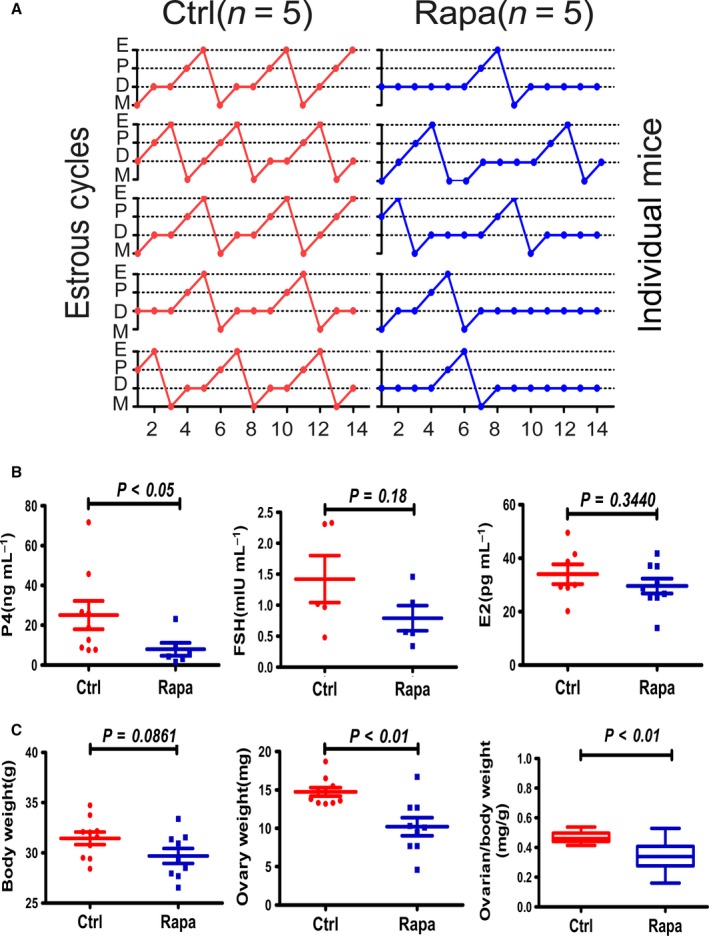
Rapa treatment disrupted physiologic functions of the ovary. (A) Vaginal smear assays were conducted daily to monitor estrous cycles during Rapa treatment. The graph shows representative cycles from the two groups (*n* = 5 per group). Each box indicates one animal, and dots represent a day. M, metestrus; D, diestrus; P, proestrus; E, estrus. (B) Analysis of serum concentrations of P4, FSH, and E2 as measured by RIA. Serum was collected immediately after 2 weeks of Rapa injection (*n* = 5–9 per group). (C) Comparison of body and ovarian weights and ovarian organ index between the two groups after Rapa treatment. The data are presented as means ± SEM.

**Figure 3:**
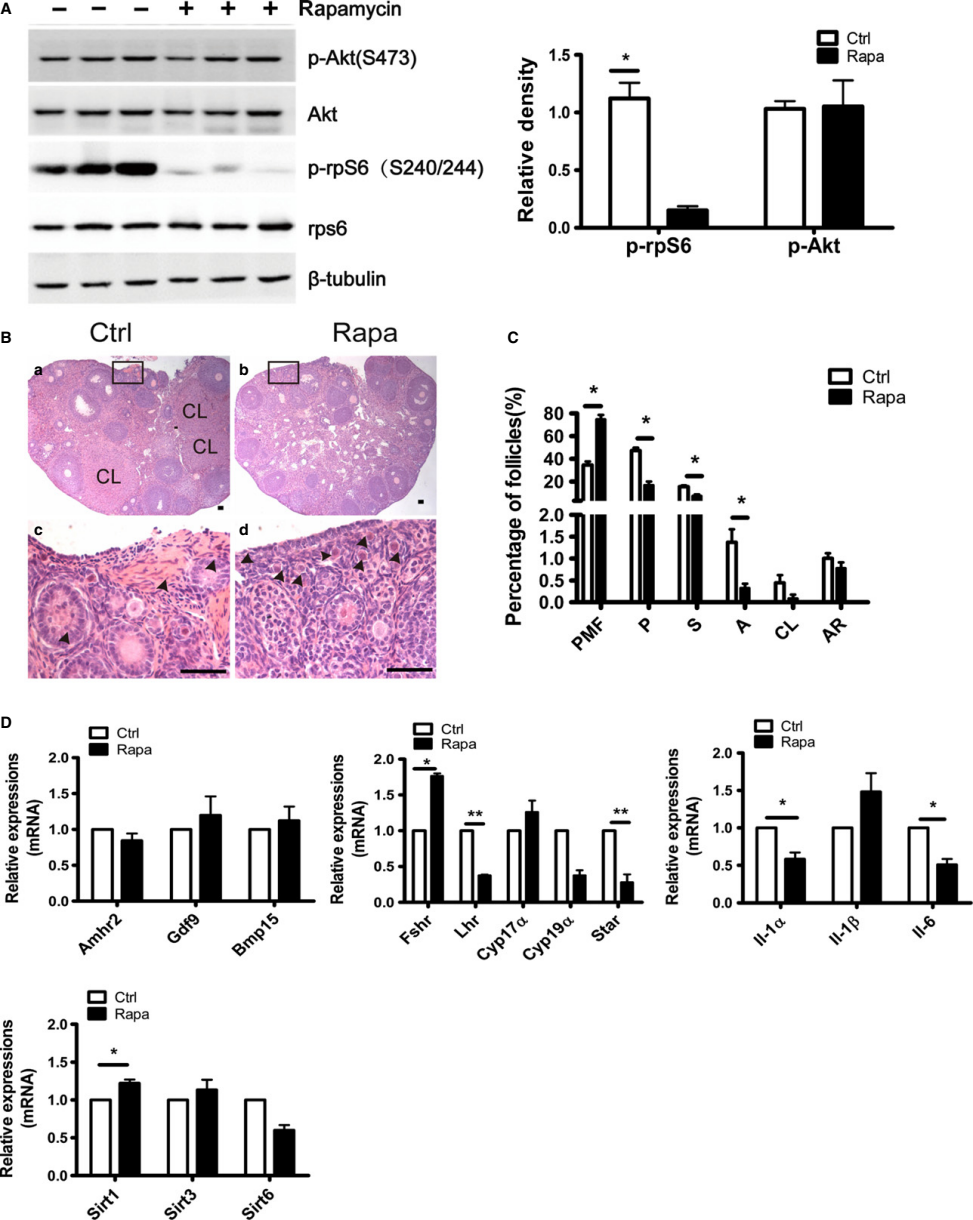
Inhibition of follicular development after Rapa treatment. Ovaries from control and Rapa‐treated mice were collected after a 2‐week duration. (A) Western blots of ovarian proteins with specific antibodies for p‐rpS6(S240/244), rpS6, p‐Akt(S473), Akt, and β‐tubulin. β‐tubulin was used as a loading control among the experimental conditions. Densitometry of Western blots was quantified and is shown by p‐rpS6(S240/2444)‐to‐rpS6 ratios and p‐Akt (S473)‐to‐Akt ratios. The data are presented as means ± SEM of three independent experiments. (B) Ovarian morphology by H&E staining. c, d are higher magnifications of insets in a and b. CL, corpus luteum. Arrow heads, primordial follicles. Bars = 100 μm. (C) Distribution of follicles at different stages per ovary (*n* = 5). PMF, primordial follicle; P, primary follicle; S, secondary follicle; A, antral follicle; CL, corpus luteum; and AR, atretic follicle. (D) Relative expression of mRNA in the ovary was assessed by real‐time PCR. Data are presented as means ± SEM. *P < 0.05 and **P < 0.01, compared with the control group.

As shown in Fig. 1, we first established a short‐term treatment model with daily injections of Rapa for 2 weeks. In the 8‐weeks mouse model, treatment was started at 8 weeks and estrous cycles were monitored daily during this period. Mice from the control group exhibited regular estrous cycles of 4–5 days duration (Fig. 2A). However, the Rapa‐treated mice showed irregular estrous cycles with a longer diestrous phase. In some animals (44%), the estrous cycle was completely lost after 1 week of injection (Fig. 2A). We then collected serum to analyze levels of estrogen (E2), progesterone (P4), and follicle‐stimulating hormone (FSH) immediately after treatment; only P4 decreased dramatically compared with the other two hormones (*P* < 0.05) (Fig. 2B). In a previous study, long‐term injection of Rapa induced severe weight loss in treated animals (Zhang *et al*., 2013), but using the short‐term protocol, treated mice did not show any loss of body weight; however, the ovarian weight or organ index of the ovary was reduced significantly (Fig. 2C). Western blotting results demonstrated a dramatic decrease in phosphorylated rpS6, the mTORC1 downstream marker, whereas the phosphorylation of Akt, the downstream marker of PI3K, did not change as much (Fig. 3A). As Akt at serine 473 can also be phosphorylated by the activation of mTORC2 (Lamming *et al*., 2012), the results suggest neither PI3K nor mTORC2 signaling pathways are affected by the short‐term Rapa treatment.

The harvested ovaries from control and Rapa‐treated mice were then fixed for morphological evaluation of follicular development. Commensurate with the reduced serum P4 levels in treated mice, corpus luteum (CL) was seldom observed in Rapa‐treated ovaries (Fig. 3B, b vs. a), and together with the decrease in growing follicles, clusters of primordial follicles were clearly found in the cortex of the ovary (Fig. 3B, d vs. c). Follicle counting results revealed that the proportions of primordial follicles nearly doubled in the ovaries of Rapa‐treated mice (74.8% vs. 34.6% in control mice), which was accompanied by a significant decrease in all growing follicles and CL (Fig. 3C). Genes related to follicular development, gonadal steroid hormone biosynthesis, inflammation, and aging were then examined using RT–PCR. As shown in Fig. 3D, the follicular development‐related genes *Gdf9*,* Bmp15*, and *Amhr2* did not change, while genes related to steroid hormone synthesis, especially *Star* and *Lhr,* declined dramatically in Rapa‐treated ovaries. As these two genes play crucial roles in P4 synthesis, and LHR (the receptor for LH and HCG [human chorionic gonadotropin]) is also indispensable for ovulation (Ascoli *et al*., 2002; Manna *et al*., 2016), such declines can be explained by the decrease in serum P4 levels and anovulation after Rapa treatment. Interleukins or sirtuins that are involved in the process of aging (Minciullo *et al*., 2016; Grabowska *et al*., 2017) were also evaluated, and *IL‐1*α, *IL‐6,* and *Sirt3* showed dramatic changes after Rapa administration. Thus, the transient Rapa treatment resulted in disturbance of ovarian functions by preserving the primordial follicle pool and inhibiting follicular development, maturation, and steroid biosynthesis.

#### Fertility rebound and prolongation of reproductive lifespan in aging females

**Figure 4:**
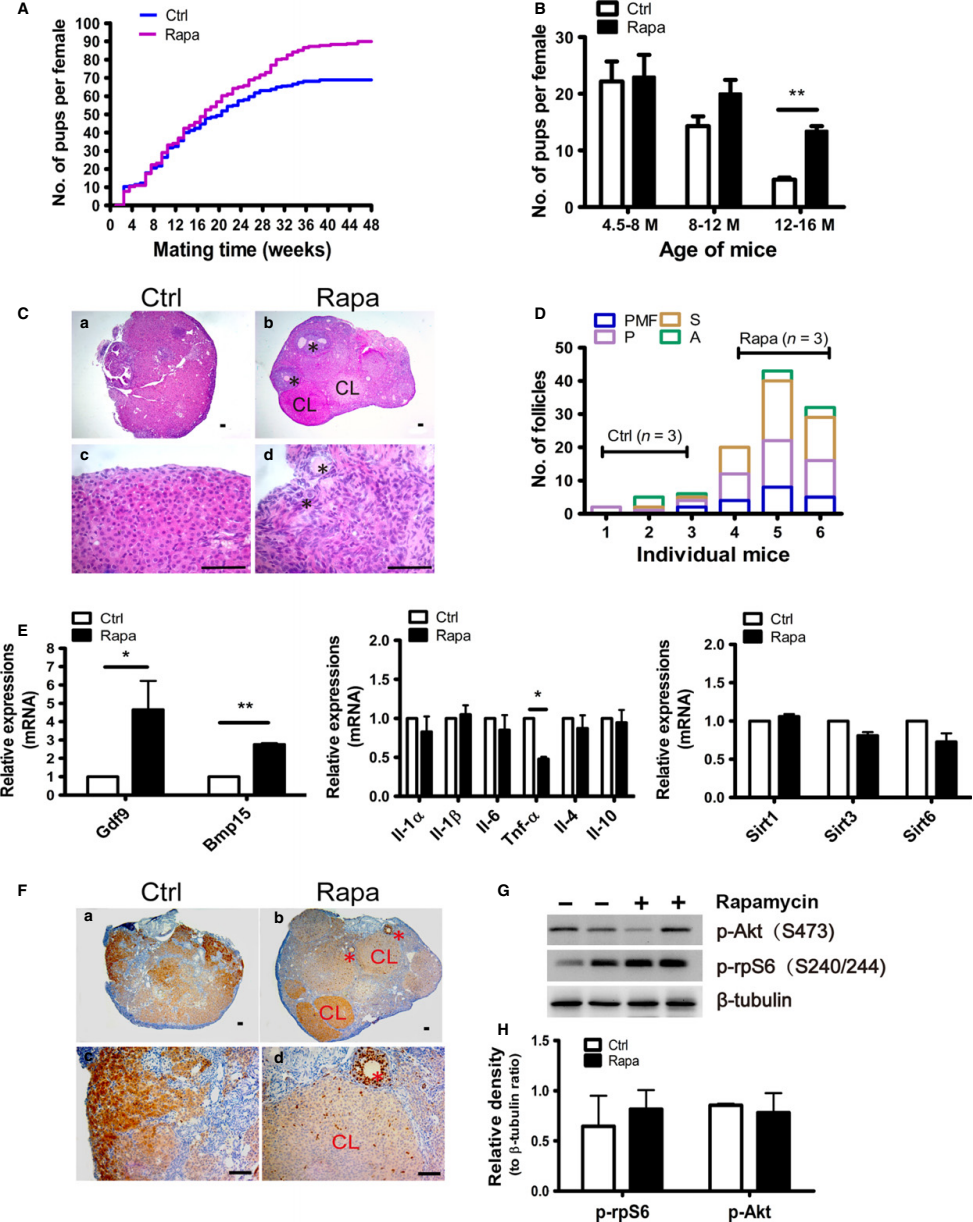
Fertility rebound and prolongation of the reproductive axis in aging females. Natural mating trials were started 2 months after cessation of Rapa treatment. (A, B) Prolongation of fertility in aging females after Rapa treatment (*n* = 24 for the control group, *n* = 23 for the Rapa‐treated group). (A) Comparison of the cumulative numbers of pups per female in control (blue) and Rapa‐treated (purple) groups. The mating trials lasted 48 weeks until mice in the control group were almost infertile at 16 months of age. (B) Results obtained from A are described for three different stages of mating trials. Mating weeks 0–16 W are equivalent to mouse ages from 4.5 to 8 months; 16–32 weeks, 8–12 months; and 32–48 weeks, 12–16 months. **P < 0.01, compared with controls. (C) Morphology of ovaries from groups with or without Rapa treatment was analyzed by H&E staining at 16 months of age. *, remaining follicles; CL, corpus luteum. (D) Follicle numbers at different developmental stages (*n* = 3 per group). PMF, primordial follicle; P, primary follicle; S, secondary follicle; and A, antral follicle. (E) Real‐time PCR of ovarian mRNAs in control and Rapa‐treated mice. Data are presented as means ± SEM. *P < 0.05 and **P < 0.01, compared with the control group. (F) Immunohistochemistry of Ki‐67 in the aged ovaries collected from control and Rapa‐treated mice. CL, corpus luteum; *, follicles. (G) Representative western blots of p‐rpS6(S240/244) and p‐Akt(S473) expression in the two groups.β‐tubulin was used as internal control. (H) Densitometry of western blots was quantified by the ratios to β‐tubulin. The data are presented as means ± SEM of 4 independent experiments.

Long‐term Rapa treatment in adult male rats has been shown to exert detrimental effects on spermatogenesis; however, such a blockade is reversible after withdrawal of Rapa treatment (Rovira *et al*., 2012). To determine whether follicular development can return to normal after Rapa treatment, estrous cycles were continuously monitored in control and Rapa‐treated groups. The recovery of ovarian function took place slowly, but 2 months later, all treated mice exhibited regular estrous cycles, with normal levels of p‐rpS6, ovarian weight and morphology, and serum hormone levels compared to control mice (Fig. S1A–D, Supporting information). RT–PCR also demonstrated the restoration of *Star* and *Lhr* mRNAs to control levels; however, *IL‐1*α, *IL‐1*β*,* and *IL‐6* still retained their lowered expression levels in treated ovaries (Fig. S1E, Supporting information). We then conducted natural mating trials in control and treated mice to test whether age‐related failure of the female reproductive axis could be postponed in Rapa‐treated mice. The mating experiment lasted 1 year, and the number of pups born from individual females was recorded to evaluate fertility. In the first several months after natural mating, the reproductive capacity in treated females (*n* = 23) was comparable to the nontreated females (*n* = 24) and pups were born healthy. After the mating trials were continued for 16 weeks and the mice were older than 8 months of age, we observed a gradual decline in female fertility in the control group, as the reproductive advantage exhibited by treated mice began to appear. The prolongation of ovarian lifespan in treated mice was most marked after 12 months of age (Fig. 4A,B). At the end of the mating trials, the females (16 months of age) in the control group exhibited a state of natural infertility whereas age‐matched mice in the Rapa‐treated group remained fertile. These results suggest that short‐term Rapa treatment started at younger age (8 weeks) prolongs ovarian lifespan in mice.

For a further assessment of follicle reserve, ovaries were collected from the two groups after mating trials ended. In control ovaries, follicles, especially primordial follicles, are very few (Fig. 4C, a and c), and the total number of follicles was less than 10 (Fig. 4D). Notably, all categories of follicles including primordial and primary follicles, as well as growing and preovulatory follicles, could be observed in ovaries of treated mice (Fig. 4C, b and d; Fig. 4D). Consistent with the ovarian morphology, mRNA levels for *Gdf9* and *Bmp15,* the two important growth factors produced by oocytes, were higher (*P* < 0.01) in treated mice. Although there were no appreciable differences in the mRNA levels for *ILs* or *Sirts*, another pro‐inflammatory factor *Tnf‐*α (which is associated with follicle loss by the apoptotic pathway (Morrison & Marcinkiewicz, 2002) was significantly reduced in Rapa‐treated mice (Fig. 4E). The expression of Ki‐67 was then used to label proliferating nuclei in control and treated ovaries, and we found that positive signals could be observed in granulosa cells of developing follicles, theca cells and corpora lutea in mice from the Rapa‐treated group; these results thereby indicated that these mice still retained normal ovarian functions (Fig. 4F). Finally, expression of p‐rpS6 (S240/244) and p‐Akt (S473) showed no obvious differences between the two groups (Fig. 4G).

#### Improvement in oocyte quality in Rapa‐treated mice

**Figure 5:**
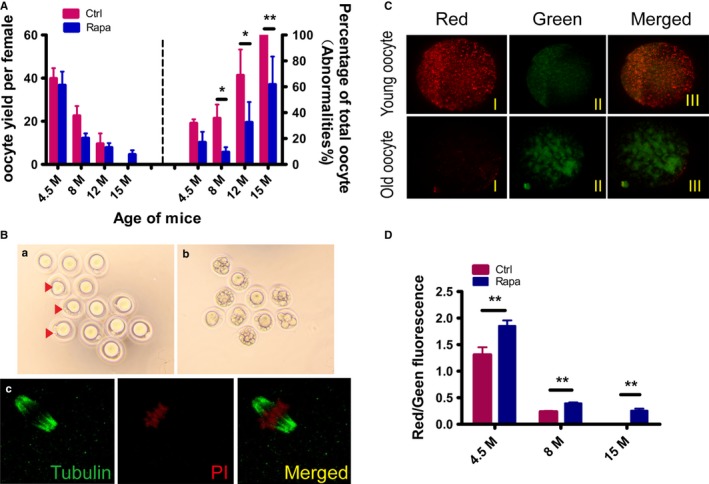
Evaluation of oocyte quality after Rapa treatment. Mice at indicated ages in control and Rapa‐treated groups were treated with PMSG for 48 h, followed by an injection of hCG, and oocytes were retrieved from oviducts 14–15 h later. (A) Comparison of oocyte yields and percentages of morphologically abnormal oocytes between control and Rapa‐treated groups. Oocytes were obtained from mice of the two groups at 4.5 months of age (4.5 months) (*n* = 6 per group), 8 months (*n* = 5 per group), 12 months (*n* = 6 per group), and 15 months (*n* = 7 in control group; *n* = 5 in treated group). As no ovulations were observed in control mice at 15 months of age, the percentage of abnormalities was designated as 100%. *P<0.05 compared with controls. (B) Morphology of oocytes obtained from Rapa‐treated female mice at 15 months: a) oocytes with normal morphology. Some showed abnormal polar bodies (red arrows). b) Oocytes with obvious cytoplasmic fragments. c) Spindle morphology by β‐tubulin (green) immunostaining of completely normal oocytes in a) red, nucleus. (C, D) Measurements of oocyte mitochondrial membrane potential by JC‐1 staining in control and Rapa‐treated groups. Oocytes were collected at 4.5 months of age, 8 months, and 15 months (*n* = 20–30 oocytes per group). (C) Representative JC‐1 staining of oocytes from young (4.5 months) and old females (15 months). Red, higher ΔΨm; green, lower ΔΨm. (D) The ratio of red/green fluorescence intensity reflects the increase in oocyte mitochondrial activity in Rapa‐treated mice. **P < 0.01 compared with controls.

We evaluated in mice the effects of Rapa treatment on oocyte quality after withdrawal of Rapa for 2 months (Age: 4.5 months), 6 months (Age: 8 months), 10 months (Age: 12 months) and 13 months (Age: 15 months). These time points are selected because mice at the above ages are representative of the gradual decline of reproductivity with age. Oocyte yield during 4.5–12 months of age was not obviously different between the two groups, and both were reduced gradually with age. However, at each stage examined, the proportions of abnormal oocytes were markedly decreased in Rapa‐treated mice (Fig. 5A). At 15 months of age, no oocytes were retrieved from control females, whereas Rapa‐treated mice still produced a certain number of oocytes, although the percentage of abnormal oocytes increased to about 60% (Fig. 5B, a and b). Morphologically healthy oocytes were then processed for spindle staining, and the oocytes exhibited normal meiotic spindles (Fig. 5B, c). In mammals, the decline in oocyte quality is related to abnormalities in mitochondrial function (May‐Panloup *et al*., 2016). Mitochondrial membrane potential (ΔΨm) is, in fact, a good indicator of mitochondrial activity and can be measured through a ratiometric analysis by JC‐1 staining. Alterations in mitochondrial membrane potential have been reported in humans with increasing age (Wilding *et al*., 2001), and, similarly, a remarkable decrease of mitochondrial membrane potential was observed in aged oocytes from both control and Rapa‐treated mice in our study (Fig. 5C,D). However, when comparing age‐matched oocytes between the two groups, the mitochondrial activity was always higher in the Rapa‐treated mice and such difference even occurred as early as at 4.5 months of age (Fig. 5D). The results suggest that Rapa treatment improve oocyte quality by increasing mitochondrial activity.

### Part II. Extension of ovarian lifespan in the 8‐months mouse model

**Figure 6:**
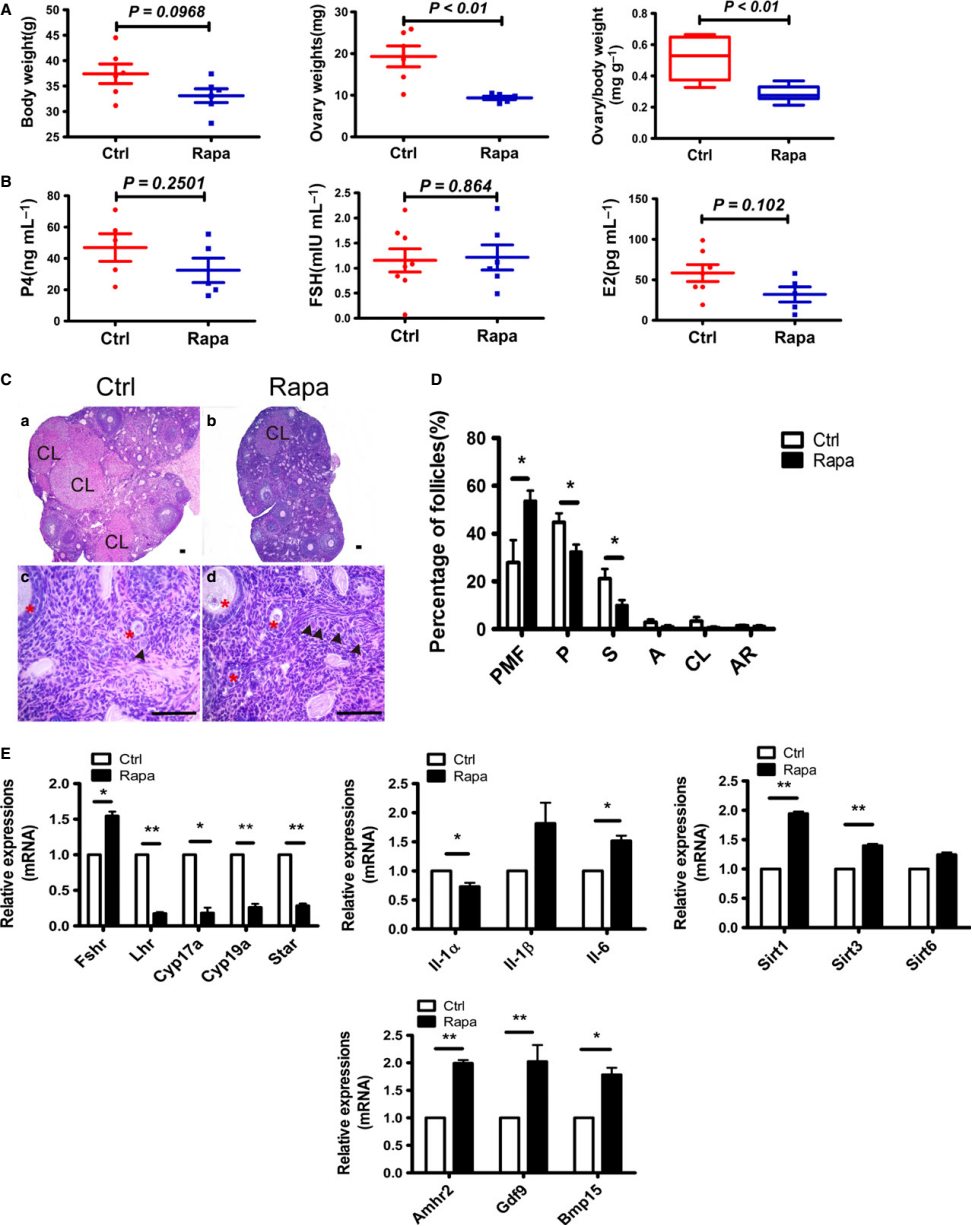
Rapa had similar effects on ovarian function in the middle‐aged (8 months) mouse model. Female mice at 8 months of age were treated with or without Rapa for 2 weeks, and ovaries were collected immediately after the injection. (A) Comparison of body weights, ovarian weights, and organ index of ovaries between the two groups (*n* = 5 per group). (B) Serum levels of P4, FSH, and E2 examined by RIA (*n* = 5–7, per group). (C) Morphology of ovaries analyzed by H&E staining. a), c) control ovaries; b), d) Rapa‐treated ovaries; and c), d) are higher magnifications of a) and b). CL, corpus luteum; arrow heads, primordial follicles. *, growing follicles. All bars = 100 μm. (D) Distribution of follicles at different developmental stages in control and Rapa‐treated groups. PMF, primordial follicle; P, primary follicle; S, secondary follicle; A, antral follicle; CL corpus luteum; and AR, atretic follicle. (E) Relative mRNA levels detected by real‐time PCR. Data are presented as means ± SEM of at least three replicates. The expression of *Actb* was used as an internal control. *P < 0.05; **P < 0.01, compared with the control group.

Although our short‐term protocol appeared to prolong ovarian lifespan in an 8‐weeks mouse model, the mice still experienced a reversible loss of ovarian function during and shortly after Rapa injection. To avoid any disturbances of young adult mouse fertility due to Rapa treatment, we next commenced treatment at 8 months of age to assess whether the prolongation of ovarian lifespan still occurred in a ‘middle‐age’ model. After termination of drug administration, similar adverse effects on ovarian function were observed in the treated mice, including a diminution in ovarian weights and inhibition of follicular development (Fig. 6A–D). The average serum concentrations of P4 and E2 showed a decreasing tendency in Rapa‐treated mice, but this was not statistically significant compared to controls (Fig. 6B). Follicle counts revealed a nearly twofold increase in primordial follicle proportions, and a significant decrease in all growing follicles (Fig. 6D). As shown by RT–PCR results, the expression of *Lhr*,* Star*, and *Il‐1*α mRNAs diminished significantly, while the expression of *Amhr2*,* Gdf9*,* Bmp15*,* Sirt1,* and *Sirt3* mRNAs increased in the treated group (Fig. 6E).

**Figure 7:**
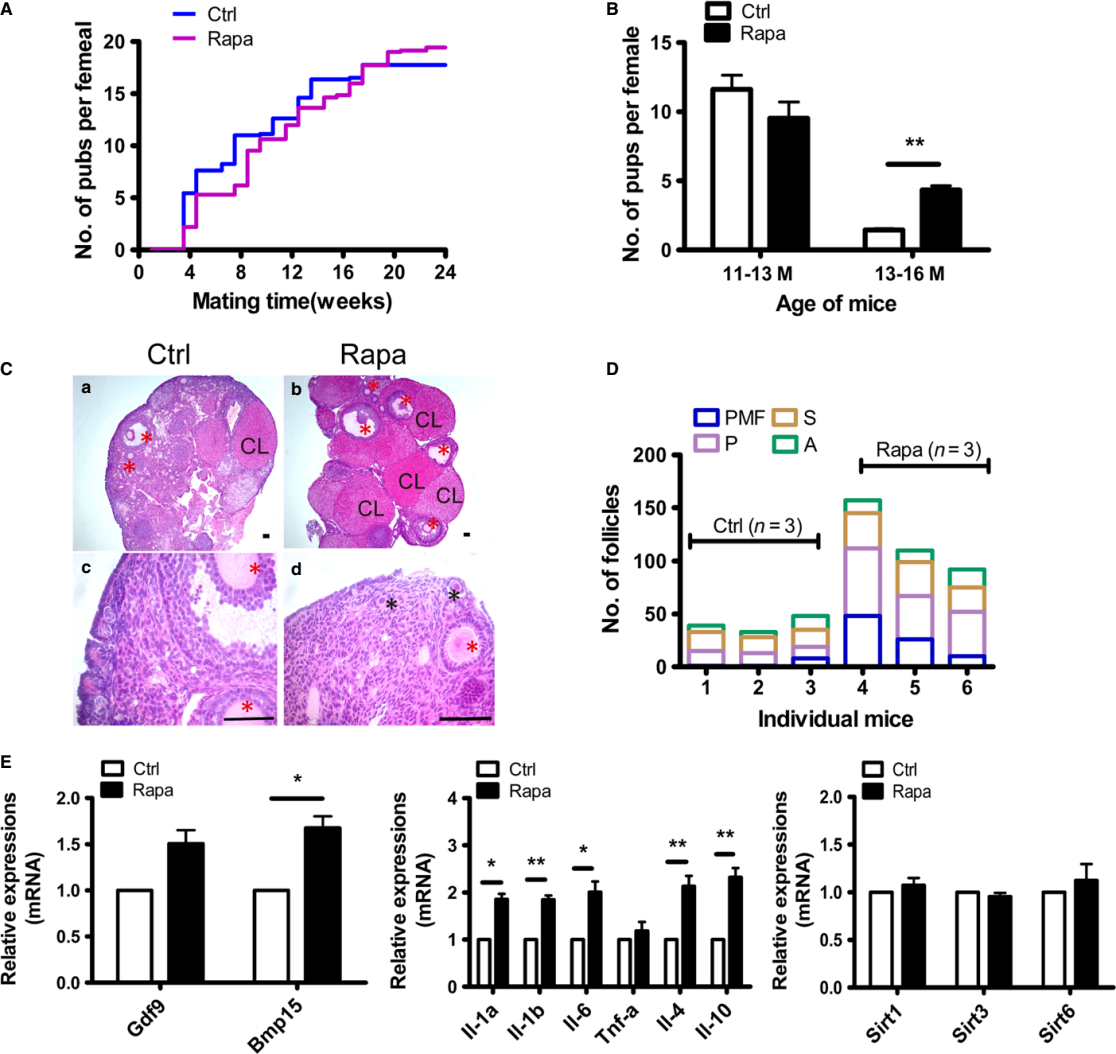
Prolongation of reproductive lifespan by Rapa treatment in the 8‐months animal model. Natural mating trials were begun 2 months after cessation of Rapa treatment. The mating trials lasted 24 weeks until mice in the control group were almost infertile at 16 months of age. (A) Comparison of the cumulative number of pups per female in control (blue) and Rapa‐treated (purple) groups (*n* = 10, per group). (B) Average number of pups per female delivered during 11–13 and 13–16 months of age. Delivered pups per female were counted independently in each stage. **P < 0.01 as compared to controls. (C) Morphology of ovaries by H&E staining. Ovaries were collected at 16 months of age after ending the mating trials. a), c) control ovary; b), d) Rapa‐treated ovary; and c), d) are higher magnifications of a) and b). red *, growing follicles; black *, primordial follicles; CL, corpus luteum. (D) Follicle counts at different developmental stages (*n* = 3, per group). (E) Real‐time PCR of ovarian mRNAs in control and Rapa‐treated groups. Data are presented as means ± SEM of at least three replicates. *P < 0.05; **P < 0.01, compared with the control group. The expression of *Actb* was used as internal control.

Natural mating trials were then undertaken 2 months later when the treated animals showed a return of normal ovarian function. The mating experiment lasted for 6 months until females were 16 months of age. As shown in Fig. 7A, after suffering a short period of fertility decline, the reproductivity in Rapa‐treated mice was quickly recovered and the advantage appeared after 12 weeks of mating. To better reflect the difference between control and Rapa‐treated mice, the mating weeks (24 weeks) were divided into two stages according to the mouse age and delivered pups were counted independently. Our data showed that in the first mating period (0–12 weeks), when mice were at 11–13 months of age, there was no difference on pups delivered per female with average 11.6 and 9.5 pups born in control and Rapa‐treated mice, respectively. In the second stage (12–24 weeks) of mating, the control mice suffered a rapid loss of their reproductive capability, bearing only an average of 1.4 pups per mouse, whereas the age‐matched females in the Rapa‐treated group still achieved an average of 4.1 pups per female mouse (Fig. 7B). As expected, after ovaries were collected at 16 months of age for histology, greater follicle numbers were preserved in ovaries of Rapa‐treated mice, especially with respect to primordial follicles, whereas this was seldom found in controls (Fig. 7C,D). Although we uncovered no differences in the mRNA levels for *Sirt1, Sirt3, or Sirt6*, expression of *Bmp15* was significantly higher. Changes in interleukin family members, including pro‐inflammatory factors *Il‐1*α, *Il‐1*β*,* and anti‐inflammatory factors *Il‐4 and Il‐10 mRNAs,* were also detected in ovaries of Rapa‐treated mice (Fig. 7E). We conclude that the short‐term protocol can prolong ovarian lifespan in 8‐months mouse model.

## Discussion

Female mammals are born with a limited number of oocytes that gradually decline during their reproductive lifespans, and in women, this continued and accelerated decline with maternal age will cause oocyte numbers to drop below 1000 at the time of the menopause (Broekmans *et al*., 2009). Compared with other species, menopause is uniquely evolved in human and occurs around 50 years of age. Although it is not as conspicuous as in human, ovarian aging is a consistent theme in mammals and many of the fundamental changes are similar, such as the loss of oocyte quantity and quality and the disruption of hypothalamic–pituitary–gonadal axis. Oocyte quality, a key component of the female reproductive axis, is also compromised with advancing maternal age, resulting in an increased risk for miscarriage and/or aneuploidy in offspring, and even infertility. Although little is known regarding the mechanism that underlies the loss of oocytes and the deterioration in oocyte quality, the follicular microenvironment is reported to play an important role in overall follicular development. Therefore, altering the properties of some of the cellular and molecular factors in the environmental milieu can change the developmental fate of oocytes, including effecting oocyte aging (Tatone *et al*., 2008). In order to sustain or increase ovarian function and improve reproductive capacity in aged women, it is then necessary not only to maintain the quantity and quality of oocytes, but also to improve the ovarian microenvironment surrounding these oocytes. Various studies have focused on extending the ovarian lifespan by preserving ovarian reserve and improving oocyte quality. One of the classic protocols is moderate chronic caloric restriction (CR) initiated in rodents during adulthood (Selesniemi *et al*., 2008, 2011). However, during a prolonged duration of CR, reproductive capacity was shown to be impaired, resulting in subfertility or even infertility in CR mice (Tilly & Sinclair, 2013). Rapa is well known as an effective inhibitor of follicular activation and has been recently reported to be able to delay ovarian aging (Luo *et al*., 2013). However, one problem remains and that is that the female rats gradually lost fertility during 10 weeks of Rapa treatment (Zhang *et al*., 2013). Moreover, whether the treatment had any effect on ovarian aging was not pursued in the same study. Therefore, before Rapa can be used to promote reproductive health in women, researchers must better understand what dose of the drug and duration of treatment are most effective, and simultaneously minimize as much as possible its side effects on reproductive capacity.

In contrast to earlier studies, our work confirms that transient Rapa administration can prolong ovarian lifespan in mice, although there still remain some negative effects on reproductive function, including reduced ovarian size, irregular estrous cycles, and inhibition of follicle development. However, these side effects lasted no more than 2 months and resulted in a rapid return of fertile potential in subsequent mating trials. In the 8‐weeks mouse model, fertility tests lasted nearly 1 year and in the 8‐months mouse model, the fertility tests were followed for 6 months. We found that Rapa improved ovarian function in aged female mice (older than 12 months of age), with higher pregnancy rates and with more healthy offspring born to both models. To our knowledge, our study is the first to reveal that short‐term administration of Rapa can prolong ovarian lifespan in aged females. However, we should mention that our results may be not as dramatic as those with the CR protocol because we minimized the treatment time so as to avoid the serious impairment of fertility that occurred during chronic CR. In women, reproductive aging that is accompanied by dramatic decreases in monthly fecundity begins at a mean age of 31 years. As our ‘middle‐age model (8 months)’ also worked well in extending ovarian lifespan, we propose a new application of Rapa in regulating ovarian function for women before reproductive aging occurs, *that is,* during the premenopause. During this period, the improvement in ovarian function induced by Rapa should be reflected in the retention of some reproductive capacity and, more importantly, should help to effectively defer the (post)menopausal period (preventing perimenopausal syndromes), and thereby improve a woman's overall quality of life.

Ovarian aging is characterized by the gradual depletion of ovarian follicles and a decrease in oocyte quality, and inhibitory effects of Rapa on follicular activation have been reported recently (Adhikari *et al*., 2013; Zhang *et al*., 2013, 2017). Consistent with these previous studies, our results also showed a blockage of primordial follicle activation during Rapa treatment that was significantly greater than that of the control group. Moreover, at the end of our experiment (at 16 months of age), more follicles at different stages were observed in the ovaries of Rapa‐treated mice, indicating to us that the ability of Rapa to sustain reproductive functions in aged animals is mediated, at least in part, by maintenance of the ovarian follicle reserve. Oocyte quality plays a critical role in the gradual age‐related decline of fertility, and increased abnormalities of the nuclear genome and the deterioration of cytoplasmic quality contribute to this decrease in oocyte quality (Qiao *et al*., 2014). Similar to results from previous studies, we also showed a decrease in the number of ovulated oocytes after exogenous ovarian stimulation (Tarin *et al*., 2001). Although there was no difference observed in the numbers of ovulated oocytes, the percentage of abnormal oocytes decreased significantly in age‐matched Rapa‐treated mice. Herein, we define abnormal oocytes as manifesting morphologic anomalies, including oocytes with cellular fragmentation, with milky or dark cytoplasm, or empty zonae pellucidae. When we evaluated the morphologically normal oocytes by spindle staining, most showed nuclear maturation with a meiotic spindle typical of MII oocytes. However, the mitochondrial membrane potential that represents mitochondrial activity increased significantly in age‐matched treated mice. The ooplasm of egg cells contains a variety of molecules and organelles that are essential for oocyte development, and which could be adversely affected by aging, and of these, mitochondria are the most important due to their roles in maternal contribution to embryonic development (May‐Panloup *et al*., 2016). Indeed, the oocyte is the richest cell of the body in mitochondria, and the effects of oocyte aging on mitochondria have been observed in morphologic and functional abnormalities. Using mitochondrial staining, we detected no differences in their distribution in morphologically normal oocytes between control and treated mice (data not shown). However, mitochondrial aggregates were reported in abnormal oocytes from aged females (Tarin *et al*., 2001). The results suggest that this phenomenon is the result of oocyte deterioration, but it does not accurately reflect the quality of the oocytes showing normal morphology. Mitochondrial membrane potential may also be different, and previous studies have demonstrated alterations in mitochondrial membrane potential with maternal aging in humans and in mice (Wilding *et al*., 2001; Ben‐Meir *et al*., 2015). Therefore, mitochondrial membrane potential may represent an appropriate marker to evaluate age‐related quality changes in oocytes with normal morphology. Rapa has been shown to rescue poor development in aged porcine oocytes *in vitro,* and to exert beneficial effects on mitochondrial function and both nuclear and cytoplasmic maturation (Lee *et al*., 2014; Song *et al*., 2014). Above all, our study revealed long‐term effects of transient Rapa treatment on oocyte quantity and quality that can last until old age in treated animals.

It is well established that the development of a competent oocyte depends upon exquisite cross‐talk among all compartments of the ovary. When oocytes are exposed to an aged ovarian microenvironment, they themselves will undergo a process of reproductive aging (Tatone *et al*., 2008). Inflammatory cytokines constitute one of the most potent factors in the ovarian microenvironment that are involved in physiologic and pathologic processes within the ovary (Boots & Jungheim, 2015), and recent studies have shown effects of inflammatory conditions on ovarian reserve in women (Freour *et al*., 2012). Elevated levels of pro‐inflammatory factors, including IL‐1β and TNF‐α (tumor necrosis factor‐a), have been reported to be associated with poor oocyte quality and negative effects on IVF outcomes (Lee *et al*., 2000; Revelli *et al*., 2009; Winger *et al*., 2009). The ovarian mRNA levels of IL‐1 family members *IL‐1*α and *IL‐1*β increased with age and *IL‐1* deficiency helped to delay the age‐related exhaustion of ovarian reserve, prolonging ovarian lifespan in mice (Uri‐Belapolsky *et al*., 2014). Chronic, low‐grade inflammation is a feature of aging, and a reduction in inflammation is proposed to be one of the mechanisms by which Rapa promotes longevity and healthspan (Lamming *et al*., 2013). In the present study, the expression of some pro‐inflammatory *IL* and age‐related *Sirt* genes showed changes in both animal models after terminating Rapa treatment. However, such changes cannot be maintained for long after removal of Rapa treatment. The expression of *Sirt mRNA* was restored to control levels soon after the treatment and without any change until the cessation of mating trials. Although *IL‐1*α, *IL‐1*β*,* and *IL‐6* mRNAs were expressed at lower levels by 2 months after Rapa treatment, no differences were observed at 4 months (data not shown). The effects of short‐term Rapa treatment on the ovarian environment can be partly explained, then, by the increase in oocyte quality in age‐matched, treated mice. This may also represent a possible mechanism by which treatment does not completely block the age‐related decrease in oocyte quality.

In summary, we have shown in mice that a transient 2‐week regimen of rapamycin was safe and that it allowed for a sufficient extension of ovarian lifespan. This new protocol is also applicable to middle‐aged animals, such that it will not only delay ovarian aging but also minimize detrimental effects on reproductive function. This novel protocol may represent an innovation in the extension of ovarian lifespan and provide new directions for future clinical applications.

## Experimental procedures

### Animals

Young adult (8 weeks) and mid‐life (8 months) CD1 female mice were purchased from Beijing Vital River Experimental Animals Centre (Beijing, China). CD1 adult male mice (10–12 weeks of age) used for mating experiments were obtained from the Model Animal Research Center of Nanjing Medical University (Nanjing, Jiangsu, China). Mice were maintained on a 12‐h light: 12‐h darkness cycle and were provided with food and water *ad libitum*. Exception for mice in mating trails, others were group housed with up to five mice per cage. All experiments requiring the use of animals were approved by the Committee on the Ethics of Animal Experiments of Nanjing Medical University. Rapamycin treatment was performed as described previously (Lamming *et al*., 2012). Briefly, 8‐weeks‐ or 8‐months‐old female mice were injected i.p. with vehicle or rapamycin (2 mg kg^−1^ BW) (LC Laboratories, Woburn, MA, USA) once daily for 2 weeks. Rapamycin was suspended in 0.9% NaCl and 2% ethanol at a concentration of 0.5 mg mL^−1^ (547 μm). Animals were ultimately sacrificed at the following time points to analyze ovarian functions: finishing injections (2 weeks), returning to normal estrous cycles (2 months after removing rapamycin), and ending natural mating tests (at 16 months of age).

### Estrous cycle analysis

Vaginal smears were collected on glass slides in 10 μL of 0.9% NaCl at 07:00–08:00 h each morning. After air‐drying, samples were stained with toluidine blue O (Amresco, Solon, OH, USA) for 3–4 min, and then washed and dried. The four stages of the estrous cycle were determined as previously described by analyzing the proportion of three major cell types (epithelial cells, cornified cells, and leukocytes) (Miyazaki *et al*., 2002). Consistent cycles of proestrus, estrus, metestrus, and diestrus (4–5 days total) in mice were called ‘regular cycles’.

### Ovarian follicle counting

Ovaries were collected and fixed in 10% buffered formalin for 12 h, embedded in paraffin, serially sectioned at a thickness of 5 μm, and then stained with hematoxylin and eosin. All follicles with a visible nucleus were counted every second section (Flaws *et al*., 1997), and in aged mice (16 months), analysis was performed on every section. Follicle classification was determined by Pederson's system (Pedersen, 1970): oocytes surrounded by a single layer of flattened or cuboidal granulosa cells were defined as primordial and primary follicles; oocytes surrounded by more than one layer of cuboidal granulosa cells with no visible antrum were determined to be secondary follicles. Antral follicle possessed a clearly defined antral space and a cumulus granulosa cell layer. Corpora lutea were filled with lutein cells, and follicles were considered atretic if they contained either a degenerating oocyte, disorganized granulosa cells, pyknotic nuclei, shrunken granulosa cells, or apoptotic bodies (Flaws *et al*., 1997). The results are reported as the number of follicles counted per ovary.

Other procedures including measurements of serum hormones, fertility testing, oocyte collection and immunofluorescence, measurement of mitochondrial membrane potential, immunoblotting analysis, immunohistochemistry, real‐time RT–PCR, and statistical analysis are provided in Appendix S1 (Supporting information).

## Funding

This study was funded by the National Basic Research Program of China (973 program, 2013CB945502), the National Natural Science Foundation of China (31671202, 31371522).

## Author contributions

X.D. and J.L. designed research; X.D., Y.S., J.L., D.H., W.L., J.Z., F.K. performed research; R.W., X.P., X.D. and J.L. analyzed data; and X.D. and J.L. wrote the paper.

## Conflict of interests

The authors declare that they have no conflict of interests.

## Supporting information


**Fig. S1** Ovarian function returned to normal after 2 months of rapamycin removal.

**Table S1** Primers for RT‐PCR.

**Appendix S1** Experimental procedures.

## References

[acel12617-bib-0001] Adhikari D , Risal S , Liu K , Shen Y (2013) Pharmacological inhibition of mTORC1 prevents over‐activation of the primordial follicle pool in response to elevated PI3K signaling. PLoS ONE 8, e53810. 23326514 10.1371/journal.pone.0053810 PMC3543305

[acel12617-bib-0002] Ascoli M , Fanelli F , Segaloff DL (2002) The lutropin/choriogonadotropin receptor, a 2002 perspective. Endocr. Rev. 23, 141–174. 11943741 10.1210/edrv.23.2.0462

[acel12617-bib-0003] Ben‐Meir A , Burstein E , Borrego‐Alvarez A , Chong J , Wong E , Yavorska T , Naranian T , Chi M , Wang Y , Bentov Y , Alexis J , Meriano J , Sung HK , Gasser DL , Moley KH , Hekimi S , Casper RF , Jurisicova A (2015) Coenzyme Q10 restores oocyte mitochondrial function and fertility during reproductive aging. Aging Cell 14, 887–895. 26111777 10.1111/acel.12368 PMC4568976

[acel12617-bib-0004] Bitto A , Ito TK , Pineda VV , LeTexier NJ , Huang HZ , Sutlief E , Tung H , Vizzini N , Chen B , Smith K , Meza D , Yajima M , Beyer RP , Kerr KF , Davis DJ , Gillespie CH , Snyder JM , Treuting PM , Kaeberlein M (2016) Transient rapamycin treatment can increase lifespan and healthspan in middle‐aged mice. Elife 5, e16351. 27549339 10.7554/eLife.16351 PMC4996648

[acel12617-bib-0005] Bjedov I , Toivonen JM , Kerr F , Slack C , Jacobson J , Foley A , Partridge L (2010) Mechanisms of life span extension by rapamycin in the fruit fly *Drosophila melanogaster* . Cell Metab. 11, 35–46. 20074526 10.1016/j.cmet.2009.11.010 PMC2824086

[acel12617-bib-0006] Boots CE , Jungheim ES (2015) Inflammation and human ovarian follicular dynamics. Semin. Reprod. Med. 33, 270–275. 26132931 10.1055/s-0035-1554928 PMC4772716

[acel12617-bib-0007] Broekmans FJ , Soules MR , Fauser BC (2009) Ovarian aging: mechanisms and clinical consequences. Endocr. Rev. 30, 465–493. 19589949 10.1210/er.2009-0006

[acel12617-bib-0008] Buckler H (2005) The menopause transition: endocrine changes and clinical symptoms. J. Br. Menopause Soc. 11, 61–65. 15970017 10.1258/136218005775544525

[acel12617-bib-0009] Flaws JA , Abbud R , Mann RJ , Nilson JH , Hirshfield AN (1997) Chronically elevated luteinizing hormone depletes primordial follicles in the mouse ovary. Biol. Reprod. 57, 1233–1237. 9369192 10.1095/biolreprod57.5.1233

[acel12617-bib-0010] Freour T , Miossec C , Bach‐Ngohou K , Dejoie T , Flamant M , Maillard O , Denis MG , Barriere P , Bruley des Varannes S , Bourreille A , Masson D (2012) Ovarian reserve in young women of reproductive age with Crohn's disease. Inflamm. Bowel Dis. 18, 1515–1522. 21936034 10.1002/ibd.21872

[acel12617-bib-0011] Grabowska W , Sikora E , Bielak‐Zmijewska A (2017) Sirtuins, a promising target in slowing down the ageing process. Biogerontology 18, 1–30. 28258519 10.1007/s10522-017-9685-9 PMC5514220

[acel12617-bib-0012] Harrison DE , Strong R , Sharp ZD , Nelson JF , Astle CM , Flurkey K , Nadon NL , Wilkinson JE , Frenkel K , Carter CS , Pahor M , Javors MA , Fernandez E , Miller RA (2009) Rapamycin fed late in life extends lifespan in genetically heterogeneous mice. Nature 460, 392–395. 19587680 10.1038/nature08221 PMC2786175

[acel12617-bib-0013] Lamming DW , Ye L , Katajisto P , Goncalves MD , Saitoh M , Stevens DM , Davis JG , Salmon AB , Richardson A , Ahima RS , Guertin DA , Sabatini DM , Baur JA (2012) Rapamycin‐induced insulin resistance is mediated by mTORC2 loss and uncoupled from longevity. Science 335, 1638–1643. 22461615 10.1126/science.1215135 PMC3324089

[acel12617-bib-0014] Lamming DW , Ye L , Sabatini DM , Baur JA (2013) Rapalogs and mTOR inhibitors as anti‐aging therapeutics. J. Clin. Invest. 123, 980–989. 23454761 10.1172/JCI64099 PMC3582126

[acel12617-bib-0015] Lee KS , Joo BS , Na YJ , Yoon MS , Choi OH , Kim WW (2000) Relationships between concentrations of tumor necrosis factor‐alpha and nitric oxide in follicular fluid and oocyte quality. J. Assist. Reprod. Genet. 17, 222–228. 10955247 10.1023/A:1009495913119 PMC3455467

[acel12617-bib-0016] Lee SE , Kim EY , Choi HY , Moon JJ , Park MJ , Lee JB , Jeong CJ , Park SP (2014) Rapamycin rescues the poor developmental capacity of aged porcine oocytes. Asian‐Aust. J. Anim. Sci. 27, 635–647. 10.5713/ajas.2013.13816 PMC4093196 25049998

[acel12617-bib-0017] Li Q , Geng X , Zheng W , Tang J , Xu B , Shi Q (2012) Current understanding of ovarian aging. Sci. China Life Sci. 55, 659–669. 22932881 10.1007/s11427-012-4352-5

[acel12617-bib-0018] Liu M , Yin Y , Ye X , Zeng M , Zhao Q , Keefe DL , Liu L (2013) Resveratrol protects against age‐associated infertility in mice. Hum. Reprod. 28, 707–717. 23293221 10.1093/humrep/des437

[acel12617-bib-0019] Luo LL , Xu JJ , Fu YC (2013) Rapamycin prolongs female reproductive lifespan. Cell Cycle 12, 3353–3354. 24091532 10.4161/cc.26578 PMC3895422

[acel12617-bib-0020] Manna PR , Stetson CL , Slominski AT , Pruitt K (2016) Role of the steroidogenic acute regulatory protein in health and disease. Endocrine 51, 7–21. 26271515 10.1007/s12020-015-0715-6 PMC4707056

[acel12617-bib-0021] May‐Panloup P , Boucret L , Chao de la Barca JM , Desquiret‐Dumas V , Ferre‐L'Hotellier V , Moriniere C , Descamps P , Procaccio V , Reynier P (2016) Ovarian ageing: the role of mitochondria in oocytes and follicles. Hum. Reprod. Update 22, 725–743. 27562289 10.1093/humupd/dmw028

[acel12617-bib-0022] Medvedik O , Lamming DW , Kim KD , Sinclair DA (2007) MSN2 and MSN4 link calorie restriction and TOR to sirtuin‐mediated lifespan extension in *Saccharomyces cerevisiae* . PLoS Biol. 5, e261. 17914901 10.1371/journal.pbio.0050261 PMC1994990

[acel12617-bib-0023] Minciullo PL , Catalano A , Mandraffino G , Casciaro M , Crucitti A , Maltese G , Morabito N , Lasco A , Gangemi S , Basile G (2016) Inflammaging and anti‐inflammaging: the role of cytokines in extreme longevity. Arch. Immunol. Ther. Exp. 64, 111–126. 10.1007/s00005-015-0377-3 26658771

[acel12617-bib-0024] Miyazaki S , Tanebe K , Sakai M , Michimata T , Tsuda H , Fujimura M , Nakamura M , Kiso Y , Saito S (2002) Interleukin 2 receptor gamma chain (gamma(c)) knockout mice show less regularity in estrous cycle but achieve normal pregnancy without fetal compromise. Am. J. Reprod. Immunol. 47, 222–230. 12069389 10.1034/j.1600-0897.2002.01074.x

[acel12617-bib-0025] Morrison LJ , Marcinkiewicz JL (2002) Tumor necrosis factor alpha enhances oocyte/follicle apoptosis in the neonatal rat ovary. Biol. Reprod. 66, 450–457. 11804962 10.1095/biolreprod66.2.450

[acel12617-bib-0026] Pedersen T (1970) Determination of follicle growth rate in the ovary of the immature mouse. J. Reprod. Fertil. 21, 81–93. 5461007 10.1530/jrf.0.0210081

[acel12617-bib-0027] Powers RW III , Kaeberlein M , Caldwell SD , Kennedy BK , Fields S (2006) Extension of chronological life span in yeast by decreased TOR pathway signaling. Genes Dev. 20, 174–184. 16418483 10.1101/gad.1381406 PMC1356109

[acel12617-bib-0028] Qiao J , Wang ZB , Feng HL , Miao YL , Wang Q , Yu Y , Wei YC , Yan J , Wang WH , Shen W , Sun SC , Schatten H , Sun QY (2014) The root of reduced fertility in aged women and possible therapentic options: current status and future perspects. Mol. Aspects Med. 38, 54–85. 23796757 10.1016/j.mam.2013.06.001

[acel12617-bib-0029] Revelli A , Delle Piane L , Casano S , Molinari E , Massobrio M , Rinaudo P (2009) Follicular fluid content and oocyte quality: from single biochemical markers to metabolomics. Reprod. Biol. Endocrinol. 7, 40. 19413899 10.1186/1477-7827-7-40 PMC2685803

[acel12617-bib-0030] Robida‐Stubbs S , Glover‐Cutter K , Lamming DW , Mizunuma M , Narasimhan SD , Neumann‐Haefelin E , Sabatini DM , Blackwell TK (2012) TOR signaling and rapamycin influence longevity by regulating SKN‐1/Nrf and DAF‐16/FoxO. Cell Metab. 15, 713–724. 22560223 10.1016/j.cmet.2012.04.007 PMC3348514

[acel12617-bib-0031] Ross C , Salmon A , Strong R , Fernandez E , Javors M , Richardson A , Tardif S (2015) Metabolic consequences of long‐term rapamycin exposure on common marmoset monkeys (*Callithrix jacchus*). Aging 7, 964–973. 26568298 10.18632/aging.100843 PMC4694066

[acel12617-bib-0032] Rovira J , Diekmann F , Ramirez‐Bajo MJ , Banon‐Maneus E , Moya‐Rull D , Campistol JM (2012) Sirolimus‐associated testicular toxicity: detrimental but reversible. Transplantation 93, 874–879. 22357177 10.1097/TP.0b013e31824bf1f0

[acel12617-bib-0033] Selesniemi K , Lee HJ , Tilly JL (2008) Moderate caloric restriction initiated in rodents during adulthood sustains function of the female reproductive axis into advanced chronological age. Aging Cell 7, 622–629. 18549458 10.1111/j.1474-9726.2008.00409.x PMC2990913

[acel12617-bib-0034] Selesniemi K , Lee HJ , Muhlhauser A , Tilly JL (2011) Prevention of maternal aging‐associated oocyte aneuploidy and meiotic spindle defects in mice by dietary and genetic strategies. Proc. Natl Acad. Sci. USA 108, 12319–12324. 21730149 10.1073/pnas.1018793108 PMC3145697

[acel12617-bib-0035] Song BS , Kim JS , Kim YH , Sim BW , Yoon SB , Cha JJ , Choi SA , Yang HJ , Mun SE , Park YH , Jeong KJ , Huh JW , Lee SR , Kim SH , Kim SU , Chang KT (2014) Induction of autophagy during *in vitro* maturation improves the nuclear and cytoplasmic maturation of porcine oocytes. Reprod. Fertil. Dev. 26, 974–981. 23902659 10.1071/RD13106

[acel12617-bib-0036] Tarin JJ , Perez‐Albala S , Cano A (2001) Cellular and morphological traits of oocytes retrieved from aging mice after exogenous ovarian stimulation. Biol. Reprod. 65, 141–150. 11420234 10.1095/biolreprod65.1.141

[acel12617-bib-0037] Tarin JJ , Perez‐Albala S , Cano A (2002a) Oral antioxidants counteract the negative effects of female aging on oocyte quantity and quality in the mouse. Mol. Reprod. Dev. 61, 385–397. 11835584 10.1002/mrd.10041

[acel12617-bib-0038] Tarin JJ , Perez‐Albala S , Pertusa JF , Cano A (2002b) Oral administration of pharmacological doses of vitamins C and E reduces reproductive fitness and impairs the ovarian and uterine functions of female mice. Theriogenology 57, 1539–1550. 12054212 10.1016/s0093-691x(02)00636-2

[acel12617-bib-0039] Tatone C , Amicarelli F , Carbone MC , Monteleone P , Caserta D , Marci R , Artini PG , Piomboni P , Focarelli R (2008) Cellular and molecular aspects of ovarian follicle ageing. Hum. Reprod. Update 14, 131–142. 18239135 10.1093/humupd/dmm048

[acel12617-bib-0040] Tilly JL , Sinclair DA (2013) Germline energetics, aging, and female infertility. Cell Metab. 17, 838–850. 23747243 10.1016/j.cmet.2013.05.007 PMC3756096

[acel12617-bib-0041] Tremellen K , Savulescu J (2014) Ovarian reserve screening: a scientific and ethical analysis. Hum. Reprod. 29, 2606–2614. 25336704 10.1093/humrep/deu265

[acel12617-bib-0042] Uri‐Belapolsky S , Shaish A , Eliyahu E , Grossman H , Levi M , Chuderland D , Ninio‐Many L , Hasky N , Shashar D , Almog T , Kandel‐Kfir M , Harats D , Shalgi R , Kamari Y (2014) Interleukin‐1 deficiency prolongs ovarian lifespan in mice. Proc. Natl Acad. Sci. USA 111, 12492–12497. 25114230 10.1073/pnas.1323955111 PMC4151745

[acel12617-bib-0053] WHO (2016) World health statistics 2016: monitoring health for the SDGs, sustainable development goals. http://www.who.int

[acel12617-bib-0043] Wilding M , Dale B , Marino M , di Matteo L , Alviggi C , Pisaturo ML , Lombardi L , De Placido G (2001) Mitochondrial aggregation patterns and activity in human oocytes and preimplantation embryos. Hum. Reprod. 16, 909–917. 11331637 10.1093/humrep/16.5.909

[acel12617-bib-0044] Winger EE , Reed JL , Ashoush S , Ahuja S , El‐Toukhy T , Taranissi M (2009) Treatment with adalimumab (Humira) and intravenous immunoglobulin improves pregnancy rates in women undergoing IVF. Am. J. Reprod. Immunol. 61, 113–120. 19055656 10.1111/j.1600-0897.2008.00669.x

[acel12617-bib-0045] Zhang XM , Li L , Xu JJ , Wang N , Liu WJ , Lin XH , Fu YC , Luo LL (2013) Rapamycin preserves the follicle pool reserve and prolongs the ovarian lifespan of female rats via modulating mTOR activation and sirtuin expression. Gene 523, 82–87. 23566837 10.1016/j.gene.2013.03.039

[acel12617-bib-0046] Zhang J , Liu W , Sun X , Kong F , Zhu Y , Lei Y , Su Y , Su Y , Li J (2017) Inhibition of mTOR signaling pathway delays follicle formation in mice. J. Cell. Physiol. 232, 585–595. 27301841 10.1002/jcp.25456

