# Supplementary figures and images for: Short‐term rapamycin treatment increases ovarian lifespan in young and middle‐aged female mice
Source: Aging Cell. 2017 May 22;16(4):825–36. doi: 10.1111/acel.12617 (PMC5506398; doi:10.1111/acel.12617)

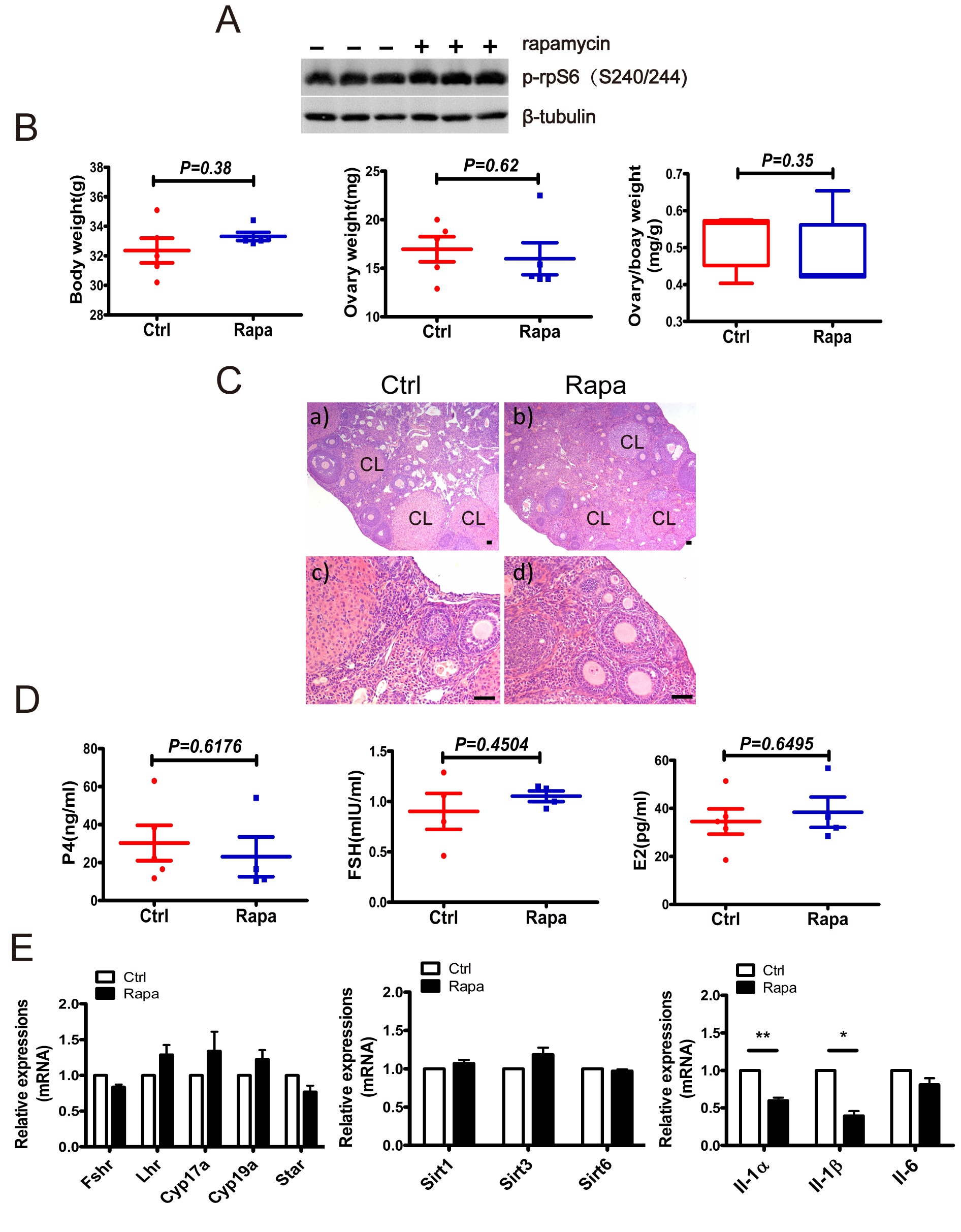

Supplement: Supplementary file 1 — Fig. S1 Ovarian function returned to normal after 2 months of rapamycin removal. [file ACEL-16-825-s001.tif]
