# Supplementary material for: Short‐term rapamycin treatment increases ovarian lifespan in young and middle‐aged female mice
Source: Aging Cell. 2017 May 22;16(4):825–36. doi: 10.1111/acel.12617 (PMC5506398; doi:10.1111/acel.12617)
Supplement: Supplementary file 2 — Table S1 Primers for RT‐PCR. [file ACEL-16-825-s002.doc]

**Table S1. Primers for RT-PCR**

| genes | Forward | Reverse |
| --- | --- | --- |
| *Actin* | 5′-CCGTAAAGACCTCTATGCC | 5′-CTCAGTAACAGTCCGCCTA |
| *Amhr* | 5′-GCAGCACAAGTATCCCCAAAC | 5′-GTCTCGGCATCCTTGCATCTC |
| *Bmp15* | 5′-TCCTTGCTGACGACCCTACAT | 5′-TACCTCAGGGGATAGCCTTGG |
| *Cyp17α* | 5′-GCCCAAGTCAAAGACACCTAAT | 5′-GTACCCAGGCGAAGAGAATAGA |
| *Cyp19α* | 5′-ATGTTCTTGGAAATGCTGAACCC | 5′-AGGACCTGGTATTGAAGACGAG |
| *Fshr* | 5′-CCTTGCTCCTGGTCTCCTTG | 5′-CTCGGTCACCTTGCTATCTTG |
| *Gdf9* | 5′-TCTTAGTAGCCTTAGCTCTCAGG | 5′-TGTCAGTCCCATCTACAGGCA |
| *Il-1a* | 5′-GCACCTTACACCTACCAGAGT | 5′-AAACTTCTGCCTGACGAGCTT |
| *Il-1β* | 5′-GCAACTGTTCCTGAACTCAACT | 5′-ATCTTTTGGGGTCCGTCAACT |
| *Il-6* | 5′-CTGCAAGAGACTTCCATCCAG | 5′-AGTGGTATAGACAGGTCTGTTGG |
| *Il-4* | 5′-GGTCTCAACCCCCAGCTAGT | 5′-GCCGATGATCTCTCTCAAGTGAT |
| *Il-10* | 5′-GCTCTTACTGACTGGCATGAG | 5′-CGCAGCTCTAGGAGCATGTG |
| *Lhr* | 5′-CGCCCGACTATCTCTCACCTA | 5′-GACAGATTGAGGAGGTTGTCAAA |
| *Sirt1* | 5′-GCTGACGACTTCGACGACG | 5′-TCGGTCAACAGGAGGTTGTCT |
| *Sirt3* | 5′-ATCCCGGACTTCAGATCCCC | 5′-CAACATGAAAAAGGGCTTGGG |
| *Sirt6* | 5′-ATGTCGGTGAATTATGCAGCA | 5′-GCTGGAGGACTGCCACATTA |
| *Star* | 5′-ATGTTCCTCGCTACGTTCAAG | 5′-CCCAGTGCTCTCCAGTTGAG |
| *Tnf-α* | 5′-CCCTCACACTCAGATCATCTTCT | 5′-GCTACGACGTGGGCTACAG |
