# Supplementary material for: Short‐term rapamycin treatment increases ovarian lifespan in young and middle‐aged female mice
Source: Aging Cell. 2017 May 22;16(4):825–36. doi: 10.1111/acel.12617 (PMC5506398; doi:10.1111/acel.12617)
Supplement: Supplementary file 3 — Appendix S1 Experimental procedures. [file ACEL-16-825-s003.doc]

**Supporting Information**

**SI Experimental Procedures**

**Measurements of serum hormones**

To avoid the influence of different estrous cycle stages on the levels of serum hormones, orbital blood was obtained between 8 to 10 am at diestrus under anesthetized conditions with pentobarbital (3 mg/100 ml, i.p.). Serum (350 μl) was separated by centrifugation at 4°C and stored at −80°C until analysis. Levels of estrogen (E2), progesterone (P4), and follicle-stimulating hormone (FSH) were measured using RIA kits provided by the Beijing North Institute of Biological Technology (Beijing, China).

**Fertility testing**

Mating trials were initiated 2 months after rapamycin cessation. Male mice at 10-12 weeks of age with proven fertility were used for mating. During the mating experiment, one female mouse was placed in a cage with one male for several months (at 16 M of age) until the mice in the control group lost their reproductive capacity. The number of offspring delivered per female was recorded once per week, and is reflected in the reproductive curve. To better evaluate the differential reproductivity between control and Rapa treated mice, we divided the whole mating weeks according to the mouse age and the number of delivered pups per female was counted independently in each stage.

**Oocyte collection and immunofluorescence**

Mice at 4.5 M, 8 M, 12 M and 15 M of age were superovulated by intraperitoneal injection of pregnant mare serum gonadotropin (PMSG, 10 IU; Sigma, St. Louis, MO, USA) followed by human chorionic gonadotrophin (hCG, 10 IU; Sigma) 46-48 h later. Oocytes were collected from oviducts 14-15 h after hCG injection. The cumulus cells were removed by pipetting in M2 medium containing 0.1% hyaluronidase (Sigma-Aldrich). Oocytes were classified as MII (with a first polar body present in the perivitelline space), maturation arrested (germinal vesicle breakdown with no polar body extrusion, or germinal vesicle intact), or degenerated (cytoplasmic fragments). For the staining of β-tubulin, oocytes were fixed in 4% paraformaldehyde in PBS buffer for 45 minutes, and then they were permeabilized with 0.5% Triton X-100 (Sigma-Aldrich, USA) for 20 minutes at room temperature. After blocking in 1% BSA-supplemented PBS for 1 h, oocytes were incubated overnight at 4℃ with anti-β-tubulin antibodies (Cell Signaling Technology, Beverly, MA, USA). After 3 washes in PBS-1% BSA, they were incubated with Alexa Fluor 488 goat anti-rabbit (Invitrogen, Carlsbad, CA, USA) for 1 h at room temperature. The nuclei were then counterstained with 0.01 mg/ml propidium iodide (Invitrogen) for 10 min.

**Measurement of mitochondrial membrane potential**

For staining of mitochondrial membrane potential, oocytes were incubated for 30 min at 37℃with 5% CO_2_ in M2 medium supplemented with 2 μM JC-1 probe (Beyotime Institute of Biotechnology, Beijing, China), in accordance with the manufacturer’s recommendations. After washing, oocytes remained in M2 and were analyzed by confocal microscopy (LSM 700, Zeiss, Germany). JC-1 exhibited potential-dependent accumulation in mitochondria, indicated by a fluorescence emission shift from green (~529 nm) to red (~590 nm). Consequently, mitochondrial depolarization was indicated by a decrease in the red/green fluorescence intensity ratio. The potential-sensitive color shift is due to concentration-dependent formation of red fluorescent J-aggregates.

**Immunoblotting analysis**

Ovarian proteins were extracted by RIPA lysis buffer (Beyotime Institute of Biotechnology) with protease inhibitor cocktails (Amresco). Proteins were then separated by electrophoresis and electronically transferred to polyvinylidene fluoride membranes. After blocking in 5% skimmed milk-TBST (TBS containing 0.1% Tween20) for 60 min, the membranes were incubated overnight at 4°C with specific antibodies. Antibodies against rpS6, p-rpS6 (S240/244), Akt (2920), p-Akt (S473), and β-tubulin were purchased from Cell Signaling Technology. Horseradish peroxidase-conjugated goat anti-rabbit and anti-mouse IgG (Zhong Shan Jin Qiao, Beijing, China) were used to detect proteins through enhanced chemiluminescence (GE Healthcare, Washington, NY).

**Immunohistochemistry**

Mouse ovaries were fixed in 10% buffered formalin for paraffin embedding and sectioning. After deparaffinization and rehydration, sections were processed for blocking of endogenous peroxidase activity and antigen retrieval pretreatment. Immunohistochemical analyses were performed using a SPlink Detection Kits (Zhong Shan Jin Qiao) with antibodies against Ki-67 (Abcam, Cambridge, MA, USA) overnight at 4°C. Negative controls were performed by incubation with non-immune rabbit IgG.

**Real-time RT-PCR**

Total RNA was isolated from 3-5 ovaries in each group by RNAprep pure Tissue Kit (TIANGEN Biotech, Beijing, China) according to the manufacturer’s instructions, and was quantified with a spectrophotometer (NanoDrop 2000c, Thermo Fisher Scientific, Waltham, MA, USA). RNA (500 ng/reaction per sample) was reverse-transcribed using a FastQuant RT Kit (TIANGEN Biotech) to create cDNA. Quantitative real-time PCR then was performed using Eva Green qPCR Master mix (Applied Biological Materials Inc., Richmond, BC, Canada) on an ABI Step One Plus platform (Thermo Fisher Scientific). The specificity of the PCR products was assessed by melting curve analyses, and amplicon size was determined by electrophoresis in 2% agarose gels. Quantification of various mRNAs was performed by using the actin amplification signal as an internal control. The specific primers used are shown in Table S1.

**Statistical Analysis**

Data are shown as means±SEM, and unpaired two-tailed Student’s *t* test was used to evaluate differences between the 2 groups. A value of P < 0.05 or P< 0.01 was accepted as statistically significant.
